# Defining a staged-based process for economic and financial evaluations of mHealth programs

**DOI:** 10.1186/s12962-017-0067-6

**Published:** 2017-04-17

**Authors:** Amnesty E. LeFevre, Samuel D. Shillcutt, Sean Broomhead, Alain B. Labrique, Tom Jones

**Affiliations:** 1 Department of International Health, Johns Hopkins Bloomberg School of Public Health, 615 N. Wolfe Street, Baltimore, MD 21205 USA; 2 Johns Hopkins University Global mHealth Initiative, 615 N. Wolfe Street E8139, Baltimore, MD 21205 USA; 3 African Centre for eHealth Excellence (Acfee), Kimberley, South Africa

**Keywords:** mHealth, Economic evaluation, Financial evaluation, Costs, Digital health

## Abstract

Mobile and wireless technology for health (mHealth) has the potential to improve health outcomes by addressing critical health systems constraints that impede coverage, utilization, and effectiveness of health services. To date, few mHealth programs have been implemented at scale and there remains a paucity of evidence on their effectiveness and value for money. This paper aims to improve understanding among mHealth program managers and key stakeholders of how to select methods for economic evaluation (comparative analysis for determining value for money) and financial evaluation (determination of the cost of implementing an intervention, estimation of costs for sustaining or expanding an intervention, and assessment of its affordability). We outline a 6 stage-based process for selecting and integrating economic and financial evaluation methods into the monitoring and evaluation of mHealth solutions including (1) defining the program strategy and linkages with key outcomes, (2) assessment of effectiveness, (3) full economic evaluation or partial evaluation, (4) sub-group analyses, (5) estimating resource requirements for expansion, (6) affordability assessment and identification of models for financial sustainability. While application of these stages optimally occurs linearly, finite resources, limited technical expertise, and the timing of evaluation initiation may impede this. We recommend that analysts prioritize economic and financial evaluation methods based on programmatic linkages with health outcomes; alignment with an mHealth solution’s broader stage of maturity and stage of evaluation; overarching monitoring and evaluation activities; stakeholder evidence needs; time point of initiation; and available resources for evaluations.

## Background

Mobile phones are the leading form of communication worldwide [1], and in many settings, access to them exceeds the availability of clean water, bank accounts or electricity [2]. Their widespread and increasing use, particularly in low- and middle-income countries (LMICs) where the disease burden is highest, has led to growing calls to harness the potential of mobile and wireless technology (mHealth) to improve health and health care delivery [3]. mHealth aims to improve health outcomes by addressing critical health systems constraints that impede coverage and utilization of health services [4]. mHealth solutions encompass a diverse range of applications of wireless and mobile technologies which may broadly be categorized into approaches focusing on (1) *health systems,* including supply chain reporting, performance monitoring, quality of care; (2) *health care providers*, including work flow management, record keeping, clinical documentation and support; and (3) *client/patient empowerment* through knowledge transfer, alerts and reminders for care-seeking [4].

Throughout the last decade, over 600 mHealth pilot strategies and programs have been introduced globally [5]. Despite the proliferation of mHealth programs, evidence on their effectiveness is still limited [6–8], with a particular dearth of economic evaluations, which aim to inform decisions on optimal resource use and allocation. To date, a small number of peer-reviewed articles comprise the body of evidence on the value for money of mHealth solutions, including cost-effectiveness analyses (CEA), cost-utility analyses (CUA), and cost-benefit analyses (CBA) [9]. While efforts to define the economics of mHealth have highlighted potential types of costs and benefits likely to emerge from program implementation [10], along with broad categories of economic evaluations available [11], guidance does not exist on which analytic approaches are most appropriate based on the maturity of the mHealth solution and/or its stage of evaluation.

Governments have found it challenging to select, scale up, and integrate mHealth solutions into existing national systems; partly due to a shortage of high quality data allowing assessments of comparative effectiveness and comparative value [12]. In this paper, we aim to improve basic understanding among mHealth decision takers, program managers, and other key stakeholders of available economic analyses to catalyse their timely and appropriate application. Improvements in the quality and frequency of the economic value of digital health strategies are, we posit, critical to their serious consideration among alternative health system investments by donors and partner governments. Over the past 5 years, our research team has provided technical assistance to dozens of large-scale mHealth programs directly and through multi- and bilateral donor agencies. Very often, although formative and summative evaluations have been planned and integrated as part of routine monitoring and evaluation, economic and financial evaluations are given scant attention. This is difficult to explain, given the seminal role this information has played in health system decision-making over the past three decades [13].

In an effort to bolster use, we outline a stage-based process for economic and financial evaluations of mHealth solutions, which aligns types of economic analyses with the stage of maturity and concomitant type of evaluation appropriate to the mHealth solution under consideration. In these stages, we distinguish economic evaluations—a form of comparative analysis for determining value for money—from financial evaluations which can be used to determine how much will be spent on an intervention, estimate the amount needed for sustaining or expanding an intervention, and compare to the amount of resources available to assess affordability. While applying these stages optimally occurs linearly and is repeated as the stage of maturity increases, we recognize that finite resources, limited technical expertise, and the timing of evaluation initiation may occur in different patterns during the implementation and policy process. We outline five steps for facilitating prioritization of which economic analyses to undertake.

### Conceptualizing a stage-based process for economic and financial evaluations of mHealth solutions

Inputs used to support health systems are called resources [14]. Costs are monetary measures of resources used to produce goods or services. Economic costs represent the value of resources used to produce a health intervention based on the concept of opportunity cost, which is the value of the next best alternative use of a resource given up when making a choice. Economic costs include both resources for which expenditures were made and those which were donated or volunteered free of charge [15], while excluding transfer payments not associated with the provision of a good or served (e.g. value added tax). Economic costs are relevant to providers as well as patients and families and in the latter instance may include productivity losses and other indirect costs. By comparison, there are two types of financial costs. One is the cash flows, the other is accrued operational and capital expenditures to purchase resources for an intervention [15]. Cash flows include money transfers from bank accounts. Accruals are expenses when they are incurred, regardless of when cash is exchanged, and expenses for which there are no cash transactions, such as depreciation, including the effect on balance sheets, and transfer payments.

Economic evaluations draw from economic costs and benefits, discounted to produce a net present value that reflects differences in the value of money over time, to determine the probable value for money of alternative resource uses. Financial evaluations use accounting costs of the resources required to implement, sustain and/or scale up an intervention. Where economic evaluations are a comparative analysis for determining what to invest in, financial evaluations help to demonstrate affordability, and estimate resource requirements for scale-up and sustainability.

**Fig. 1:**
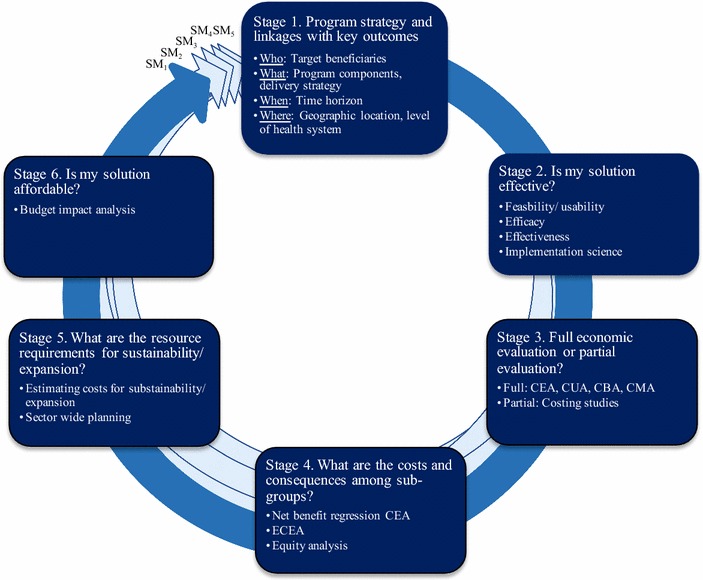
Conceptualizing a stage-based process for economic and financial evaluations of mHealth solutions. SM_1-5_ corresponds to Stages of maturity 1–5 denoting the need to repeat the stages to catalyze advancement to the next stage of maturity

Collectively, economic and financial evaluations may be conceptualized as part of a larger appraisal process. Appraisals encompass a broad framework of activities, which generate evidence necessary for decision-taking and reviews on the worth of an intervention [16]. In this context, economic and financial evaluations become critical ‘stages’ repeated with each progression in an mHealth solution’s stage of maturity from pre-prototype, prototype, pilot, demonstration, scale-up, to integration and sustainability (Fig. 1) [17]. These stages can be completed independently in succession, or as part of a larger business case, which includes concurrent efforts to outline a strategic case (context, need, anticipated outcomes and impact), commercial case (viability of supply side), and management case [arrangements for program delivery, including monitoring and evaluation (M&E)] [16, 18, 19]. In the text to follow, we review each Stage in turn and then outline a 5-step process for facilitating decision making on which analyses are most appropriate for a given mHealth program strategy based on its stage of maturity and evaluation, timing of initiation and available resources.

### Stage 1. Define your program strategy and linkages with key outcomes

Defining the mHealth program strategy is a vital starting point for economic and financial evaluations, and one which will require re-assessment continuously as programs mature and grow over time. Efforts to define the program strategy should outline the ingredients which underline the program, including who (key stakeholders, target population and characteristics), what (program components, delivery strategy, activities required to develop, start-up and maintain implementation), when (time period), and where (geographic location, level of health system) implementation is occuring. Once the program strategy has been defined, the pathway between this strategy and a target outcome must be defined. While we consider broader issues around the monitoring and evaluation of mHealth programs in greater depth elsewhere [17], in brief, this can be done through the application of a theory of change [20], results framework [21], logic-model [22], or other conceptual model^1^ [23]. As part of this process, analysts should consider three critical factors: (1) does the mHealth solution have a direct effect on health status or does it aim to enhance the delivery of an intervention with known effectiveness? (2) are sub-group differences in the uptake of the mHealth solution anticipated? (3) is the mHealth solution anticipated to influence the broader financial wellness of the household? The answers to these questions will influence the type of evaluation study designs, outcome/impact level data available, and ultimately the types of economic analyses which are feasible to conduct.

For mHealth solutions aiming to have a direct impact on health, a more rigorous process of impact evaluation may be indicated starting with efficacy and effectiveness studies. By comparison, for mHealth solutions aiming to improve delivery of an intervention with a well-established evidence base, emphasis may instead be on the indirect relationship between the mHealth solution and health outcomes. In such instances, the evidence base for health effects, may render the measurement of health outcomes to be unethical or unnecessary. The emphasis may instead be placed on the effectiveness of the delivery strategy as a catalyst for improving service delivery or utilization, therefore restricting the type of outcome measure available for use in economic analyses to changes in coverage, or process indicators. For these mHealth programmes, opportunities should be explored for translating coverage data for key reproductive, maternal, newborn, and child health (RMNCH) interventions into modelling tools such as the Lives Saved Tool (LiST) [24] to generate estimates of lives saved for individual and packages of interventions. For mHealth solutions which are anticipated to have a differential affect across sub-groups, opportunities for collecting data on financial protection, equity, and out of pocket spending should be explored from the outset. For mHealth solutions which are anticipated to have an impact on the financial wellness of the broader household, efforts need to be undertaken from the outset to additionally measure these and ensure their inclusion.

### Stage 2. Is my solution effective?

Methods for determining the effectiveness of mHealth solutions are well-established and several guidelines exist to facilitate their design and implementation [17]. Efforts to determine the effectiveness of an mHealth solution play a vital role in defining the scope, utility, and feasibility of conducting economic and financial evaluations. As part of efforts to determine the effectiveness of an mHealth solution, it is important to understand what role the mHealth solution has in catalysing changes in process, performance or health effects. Where an mHealth solution has a direct effect on health outcomes, data on health outcomes may be measured. Where the mHealth solution aims to improve delivery of an intervention with known effectiveness, quantifying the direct effect of the mHealth solution on health outcomes may not be required and outcome measures for coverage, changes in service delivery, practices or efficiency may be used in their existing form or to model changes in health outcomes including lives saved.

If an mHealth solution is not effective in a given context according to pre-defined objectives, efforts to determine its value for money, and/or affordability may be contraindicated. In some instances, even improvements in worker satisfaction, a commonly cited indicator of early-stage digital health success, may be used as an outcome metric, provided the investor in the solution is interested in improving satisfaction. If an mHealth solution is effective, the intended audience, study design, available data, policy time frame, proficiency of the analysts, disease epidemiology, and emerging results may drive the selection of economic, then financial evaluations methodologies. Effectiveness studies which adopt randomized or quasi-experimental study designs, and have data on the costs and consequences of two or more alternatives, may enable economic evaluations to be conducted based on primary data. However, when only data on a single program are available and a comparator or alternative cannot be modelled, partial evaluations or costings studies may be all that are feasible.

### Stage 3. Full economic evaluation or partial evaluation?

Full economic evaluations compare two or more alternative courses of action in terms of both their costs and consequences [25]. This category of study includes CEAs, which use natural, identical units to measure changes in outcomes; CUAs which measure outcomes in terms of utility measures such as Quality Adjusted Life Years (QALYs) or Disability Adjusted Life Years (DALYs); CBAs, where both inputs and outcomes are evaluated in monetary terms; and cost-minimization analysis (CMA) which assumes equivalent outcomes before the study onset [26]. Despite differences in the valuation of consequences or measures of effect, full economic evaluations value resources in similar ways. Differences in the valuation of consequences reflect the different aims and viewpoints of different decision problems. Where consequences have been designed to demonstrate equivalence, a CMA may be used to determine the lowest-cost mHealth solution. However, appropriate applications of CMA are rare [27, 28], and far more common are CEAs, CUAs, and CBAs. For mHealth studies which can monetize outcomes, CBAs may be a useful tool for making comparisons across economic sectors, or for mHealth solutions whose benefits include productivity and efficiency gains that result in direct or measurable health gains for a wide range of stakeholders. For studies which do not monetize consequences, CEAs and CUAs are more appropriate analytic tools. CUA’s utility measures which consider both length of life and subjective levels of well-being, can provide a more comprehensive picture of health status and allow for comparisons across programs and disease areas. In CEAs the effects of the interventions are measured in identical units of outcome and can only be compared against alternatives with the same outcome (e.g. number of children exclusively breastfed or number of lives saved).

**Fig. 2:**
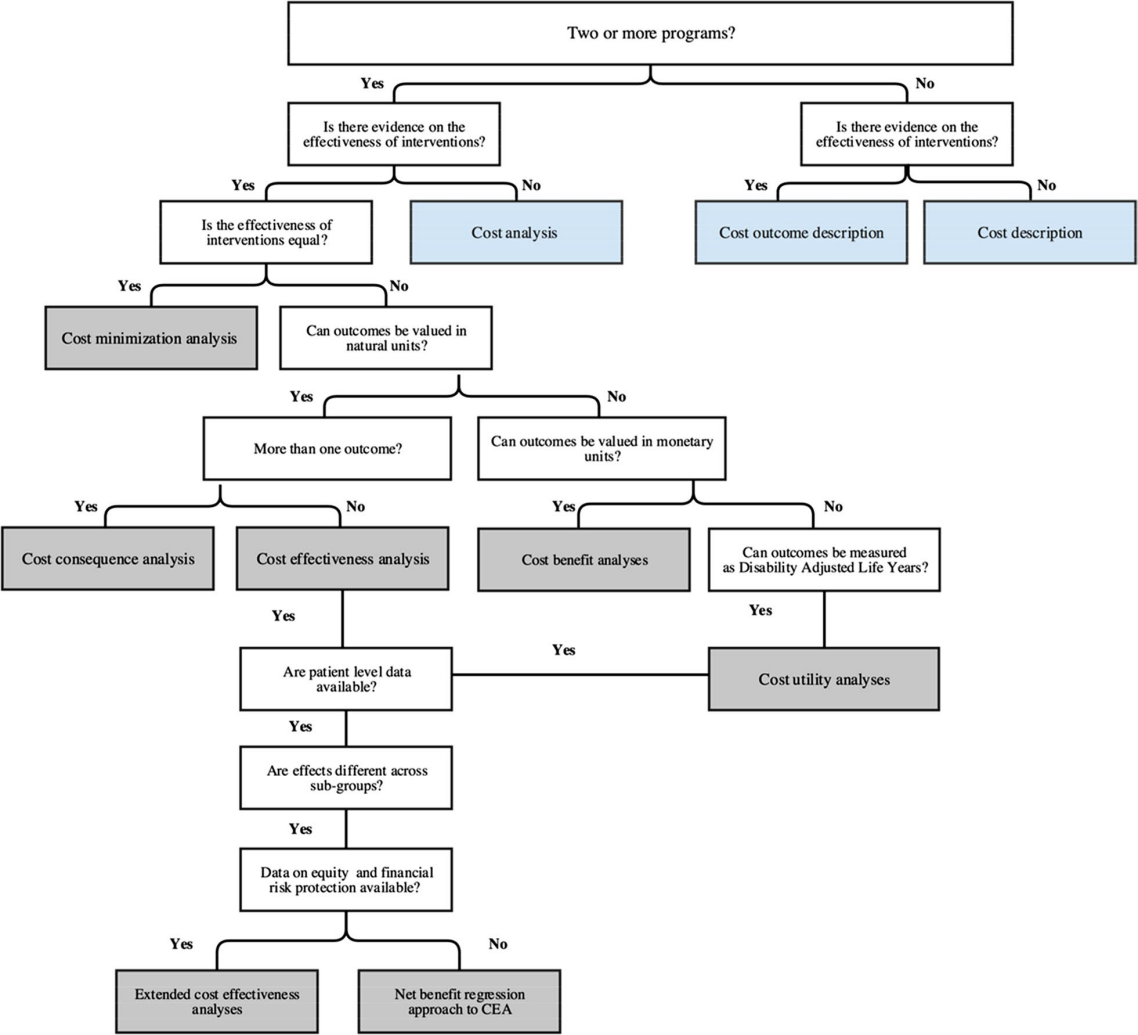
Choosing between alternative types of economic evaluations (adapted from [53–55])

Where programs do not have the data, resources, time or expertise to conduct a full economic evaluation, partial evaluations (sometimes referred to as ‘costing studies’) may be undertaken to measure the costs of a single program (cost description); to measure costs of a program and alternatives (cost analysis); or to describe the costs and consequences of a single program (cost outcome description analyses) [13]. While partial evaluations do not make explicit comparisons in the costs and consequences of alternatives [29], they can provide useful insights for stakeholders by identifying potential amounts of costs, understanding key drivers of costs, and/or preparing to estimate the resources required to sustain or expand a solution, or to develop more comprehensive economic evaluations [30]. Table 1 summarizes the most common forms of financial and economic evaluations. Figure 2 presents a flow chart for facilitating decision-making on which type of economic evaluation is indicated based on the data available.

**Table 1:** Types of economic evaluation for mHealth

	Definition	Costs	Consequences	Primary audience
Economic evaluations				
Cost effectiveness analysis	Comparison of two alternatives where consequences of the programme are measured in natural units	Monetary units	Natural units (life years gained, lives saved, cases detected)	Decision-takers within a speciality
Cost utility analyses	Form of CEA where consequence is measure in terms of healthy years	Monetary units	Summary measure of population health: QALYs or DALYs	Health sector government NGO Decision takers across sectors
Cost benefit analysis^a^	Comparison of two alternatives where consequences of the programme are measured in monetary terms.	Monetary units	Monetary units	Decision takers across sectors
Extended cost effectiveness analysis	Form of CEA which assesses both the financial risk protection (catastrophic health costs averted, cases of poverty averted, money-metric value of insurance) and the equitable distribution of costs and health gains across population sub-groups and to policy makers	Monetary units	Health gains garnered across population sub-groups; may also use a summary measure of population health (QALYs or DALYs)	Health sector government NGO Decision takers across sectors
Cost consequence analysis	Form of CBA which examines costs and consequences but does not aggregate consequences into a single measure	Monetary units	Natural units	Decision takers across sectors
Cost minimization analysis	Compares relative costs of interventions with effects that are assumed to be equivalent	Monetary units	Assumed to be equivalent	Decision-takers within a speciality
Partial evaluations				
Costing analysis	Comparison of the costs of two or more programs	Monetary units	Not applicable	Decision-takers within a speciality
Cost description analysis	Describes the costs of a single program	Monetary units	Not applicable	Decision-takers within a speciality
Cost outcome description analysis	Describes the costs and consequences of a single program	Monetary units	Natural units (life years gained, lives saved, cases detected)	Decision-takers within a speciality
Financial evaluations				
Financial forecast model	Estimates the financial and accounting profile of the capital and operational cash flow and income and expenditure of a project option over its whole life-cycle for comparison with budgets and financial plans to seek affordability	Monetary units	Not applicable	Reimbursement authorities
Budget impact analysis	Estimates the financial consequences of an intervention and its diffusion within a specific health-care setting or system context given resource constraints	Monetary units	Number of beneficiaries or affected individuals	Reimbursement authorities
OneHealth	National strategic health planning tool which provides analysts with a single framework which includes scenario analysis, costing, health impact analysis, budgeting and financing of strategies for all major diseases and health system components. Outputs facilitate (1) the identification of resource needs to implement a strategic health plan; (2) the determination of costs for the strategic plan by year and input; and (3) estimates of health impact [49]	Monetary units	Natural units (lives saved)	National planners

^a^One type of CBA conducted for regulations instead of interventions is called “regulatory impact analysis” which can influence how networks are regulated. Applications of this may be appropriate for eHealth, particularly to inform decision making on how best to develop regulations on the interoperability of health information infrastructures and data protection [56]

### Stage 4. How do costs and consequences vary across key population sub-groups?

Variability in population responses to an mHealth solution may correspond to heterogeneity in program uptake and the health outcomes observed. Partitioning patient, provider or citizen populations into sub-populations, communities or groups and assessing the expected costs and consequences on these groups can facilitate decision-taking on the optimal allocation of resources. Given finite resources, the differential allocation of resources to different target populations may yield greater improvements in overall health gains [31].

For mHealth programs expected to yield differences in costs and consequences across sub-groups, CEAs using a net benefit regression framework (NBRF) may be appropriate. Applications of NBRF marry econometrics with CEA or CUAs to improve the handling of uncertainty, control for confounding, and “allow analysts to explore the importance of covariates on the marginal cost effectiveness of an intervention” (i.e., interaction effects between the intervention and important subgroups)” [32]. In common applications of CEAs or CUAs, incremental cost effectiveness ratios (ICERs) are generated which compare the expected values of cost and consequences across alternatives. An intervention is deemed good value for money if its ICER is below a pre-determined maximum willingness to pay for health gain. In practice, since two different treatments or interventions cannot be applied to the same population simultaneously, the true incremental costs and true incremental consequences of an intervention remain unknown [32]. Drawing upon sample data obtained from clinical trials or programs, it is possible to estimate the sample mean and sample consequences of a true but unobservable ICER parameter. However, using ratio statistics may pose statistical problems which may affect the interpretation of sampling uncertainty in the ICER [33, 34]. Since ICERs are not amenable to regression-based methods, the marginal effect of an intervention on key population sub-groups (e.g. gender, ethnicity) cannot be determined while controlling for other co-variates [32].

In NBRF, the traditional equation which divides changes in sample mean costs (ΔC) by changes in mean effects (ΔE) to generate an (ΔC/ΔE) ICER is re-arranged by multiplying each arm of the equation by ΔE [34, 35]. The result is ΔC = ΔE * ICER and for any ceiling ratio λ, ΔC = ΔE * λ. In NBRF, the dependent variable is calculated as a net monetary benefit statistic with the equation ΔE * λ − ΔC = NMB. When computed at an individual level, the resulting equation of NB_i_ = ΔE_i_ * λ − ΔC_i_ mirrors that of simple linear regression equation Y = α + β*x*_i_ + ε_i_ where Y is the dependent variable, α is the y-intercept, β the regression coefficient on an explanatory variable, and ε_i_ is the standard error [35]. The basic NBRF model can then be expanded to include important covariates and interaction terms, to control for confounding and evaluate incremental cost effectiveness for those subgroups [32]. NBRF is strategy which improves not only the handling of uncertainty in economic evaluation, but allows analysts to identify and account for important determinants that affect the cost-effectiveness results [32, 34]. NBRF also has the added advantage of allowing analysts to model different probabilities that an mHealth solution would be preferred over alternatives given different budget constraints [35].

To estimate the differential affect across sub-groups, analysts can consider dimensions of equity and financial protection. In many countries, out of pocket expenditures for health care, particularly when added to income loss from illness, are leading causes of impoverishment [36, 37]. While some economic evaluations may assume the perspective of the user and aim to measure out of pocket payments for care, they are not principally concerned with measuring catastrophic spending, nor do they consider non-health or wider economic and social benefits of investing in a particular innovation [38]. Extended cost effectiveness analysis (ECEA) has emerged as a tool for synthesizing the health and financial risk protection benefits and distributional consequences of policy [36, 38]. Building on standard CEA results on costs per unit of health gain, ECEAs assess both the financial protection (including cases of catastrophic health costs averted, poverty cases averted, and money metric value of insurance) and the equitable distribution of costs and health gains across population sub-groups. While there remains a paucity of applications of ECEAs in the literature, and none to date for mHealth, benefits for mHealth solutions could be evaluated across dimensions of direct out of pocket payments for medical and non-medical costs; financial risk protection^2^; health benefits (lives saved; hospitalizations averted); and distributional consequences across sub-groups [36]. ECEA applications to date have explored financial protection and the health gains garnered across population sub-groups defined by socioeconomic status (wealth quintiles). ECEA could also explore differences across other dimensions of equity including gender, ethnicity, geography, and education given the disparities in health or in social determinants of health between social groups who have different levels of underlying social advantage/disadvantage [39, 40]. ECEA is most suited for evaluating health benefits, financial risk protection, total costs to stakeholders, and equity in one analytic framework.

### Stage 5. What are the resource requirements for expanding delivery?

Analytical frameworks [41], tools [42], and methods to support the financial planning and the successful expansion of mHealth solutions are emerging [43–45]. Health systems frameworks for scaling up mHealth solutions recommend that financial planning occurs as a part of a larger planning for scale process which additionally includes groundwork, a review of policies, partnerships (including government stewardship), technology and architecture systems, operations, and M&E [41, 43, 44]. While there are many approaches to financial planning for scale, two starting points are to: (1) develop a financial forecast for sustaining and/or expanding program activities; and (2) model the sector wide implications of scaling up.

Financial evaluations that estimate the costs to deliver an mHealth program at scale may start by estimating the total addressable market, revenue streams, total cost of ownership, and ultimately explore break-even scenarios before identifying a preferred strategy for scale. Efforts to determine the total addressable market will start with detailed planning on how and where program rollout will occur and at what pace, and include estimates of the total number of beneficiaries. This may be followed by efforts to identify revenue streams, including the willingness to pay on the part of key customer segments (where appropriate). Once the beneficiary population and revenue streams have been identified, context specific adaptations required in response to local epidemiology, population and health systems needs as well as connectivity and infrastructure should be identified. Understanding that multiple strategies for scaling up a particular program exist, it may be advisable to develop several expansion scenarios. Once program specifications and scenarios are finalized, drawing from primary data, capital and operational costs to modify/develop, start-up, and maintain program implementation should be estimated for each scenario as part of efforts to determine the total cost of ownership [46, 47]. While the analytic time horizon and perspective taken will depend on the intended audience and implementers, costs for most mHealth program should seldom exceed a 5-year analytic time horizon in light of the rapid pace with which technology changes, and the depreciation inherent to technology investments. Once total cost of ownership has been determined, a breakeven analysis can be conducted to facilitate decision-making on the preferred strategy for scale. Examples of breakeven analyses are emerging in the literature [48] as one tool for facilitating understanding of the optimal strategy and its growth trajectory in conjunction with costs and sources of probable revenue. Beyond facilitating decision-making on the optimal scale up scenario, such analyses may help implementers to negotiate with key stakeholders, including mobile network operators to reduce costs and/or identify the optimal cost recovery strategy required to ensure long-term sustainability.

Once the preferred strategy for expansion/sustainability is identified, the broader sector-wide implications of integrating an mHealth program into the exciting health system should be considered. Depending on the mHealth solution, programmatic context and scale, the OneHealth tool may be an appropriate alternative tool. Designed to inform national strategic health planning in LMICs, OneHealth provides analysts with “a single framework for scenario analysis, costing, health impact analysis, budgeting and financing of strategies for all major diseases and health system components” [49]. Outputs facilitate (1) identifying resource needs for strategic health plans; (2) estimating costs for strategic plans by year and input; (3) estimating health impacts [49]. Based on these outputs, analysts can then compare costs with available finance [49]. Based on these outputs, analysts can then compare costs with available finance [49].

### Stage 6. Is my mHealth program strategy affordable?

Affordability measures the extent to which net accounting costs and cash flow match the provisions in annual budgets and financial plans over a specified time period. Affordability should be assessed at multiple time points in the stage-based process through three major financial statements: (1) budget statement (draws from resource accounting and budgeting to illustrate resources over the lifetime of a proposal); (2) cashflow statement (depicts the additional cash flow needed if the lead option goes forward); and (3) funding statement (shows resources slated for provision from key stakeholders) [16]. Affordability is met if the following six criteria are met: (1) the balance sheet correctly accounts for assets and liabilities, and (2) it is healthy; (3) the organization or service unit is solvent; (4) it is not overtrading; (5) the cash flow of the organization is sound; and (6) allowances for risk have been made [16]. For unaffordable mHealth programs, either the scope of activities underpinning the mHealth project costs need reducing; the scale of implementation changed; and/or the overall budget needs increasing. Given that changes in the design of an mHealth program could have implications on both costs and consequences, options for affordability should be iterated with the value for money estimates generated by economic evaluations to find an optimal relationship between them that guides final investment decisions. Setting an affordability plan in place may also require an assessment of alternative financing models and their sustainability. This could entail identifying new collaborative partnerships to facilitate cost-sharing; negotiating with key vendors to reduce costs (e.g. mobile network operators to reduce airtime costs); and/or exploring alternative service delivery models (e.g. ‘freemium models where users pay for some features) [43]. Where these change an mHealth program’s resource profile too, further refinements to value for money analyses may be required.

To assess affordability, the Bill and Melinda Gates Foundation, NICE International, and other stakeholders recommend conducting a Budget Impact Analysis (BIAs) [31]. While BIAs can be performed in isolation, they are most commonly presented with CEAs and used to estimate the financial consequences of adopting a new intervention [50]. This falls in contrast to CEAs which aim to measure the relative value of an intervention against an alternative; ultimately generating a measure of the *additional* cost per outcome measure [51]. BIAs typically assume a 1–5 year analytic time horizon, adopt the perspective of the budget holder, and present the financial streams for each budget period of interest as undiscounted costs [50]. Most BIAs will exclude overhead costs and measure only the direct costs of inputs required to implement the intervention [51]. Additional BIA reporting standards can be found in country-specific and global guidelines [50]. Table 2 outlines differences in the content of steps for conducting BIAs alongside full economic evaluations and partial evaluations.

**Table 2:** Differences in the content of steps for conducting economic and financial evaluations of mHealth solutions

Steps	Full economic evaluation	Costing study	Cost description analysis	Cost outcome description analysis	Budget impact analysis	Financial forecasting for scale
Define the objective	Identify the research questions, define the purpose of the work, audience for and intended use of information; Objective should clearly state the perspective, population, locations, time horizon, and for full economic evaluation, the comparator and outcomes selected					
Define the perspective or viewpoint from which the analysis is undertaken	Societal perspective is considered gold standard; collection of all perspectives allows for scenarios to be presented for individual perspectives (program, user, payer, health systems)	Program perspective^a^ most common			MEEP: Societal perspective [31] ISPOR: Program perspective	Program perspective
Define the intervention	Target population size and characteristics, Target disease(s), Delivery sites, Technology and architecture systems, Activities required to develop, start-up and maintain implementation					
Define the comparator against which costs and effects are measured	Minimally the following comparators should be assessed: Interventions/program or mix currently available to the population The most cost-effective alternative ‘do nothing’ analysis representing non-interventional care for the population [31]		Not applicable; single program		Minimally the following comparators should be assessed: Interventions/program or mix currently available to the population The most cost-effective alternative ‘do nothing’ analysis representing non-interventional care for the population	Not applicable; single program
Define the time horizon, including the *time frame* (period in which intervention is applied) and *analytic time horizon* (period for which costs and consequences are considered) [57]	*Trial based evaluations* Time frame aligned with program duration *Model based* Defined by analyst should be of sufficient length to capture all costs and effects relevant to the decision problem				Time horizon of relevance to the budget holder; 1 to 5 years is most common but may vary by country/organization and therefore reasons for choice should be stated	
Identify, measure, and value consequences	Measure of health outcome specific to the decision problem; should capture positive and negative effects on length of life and quality of life; should be generalizable across disease states [31] CBAs may include non-health outcomes Many studies may include synthesis estimates if effects on multiple target groups are anticipated Since results may be sensitive to outcome, it may be useful to test several	Not applicable		Measure of outputs/health outcome specific to the decision problem; should capture positive and negative effects on length of life and quality of life; should be generalizable across disease states		Impact on health outcomes may be forecasted
Identify, measure, and value costs	Use of economic costs or approximations Primary vs secondary data source Data reported on expected resource use and costs of delivery to the target population(s) Overall costs of interventions reported as well as costs of resource inputs Categorization of costs: capital vs. recurrent, fixed vs. semi variable vs. variable costs Annualization of capital and fixed costs over the period of implementation [31] Discounting/constant prices Inflation to base year Converting costs into USD For some analyses, converting to international dollars with purchasing power parities may also be indicated	Use of financial costs most common Data reported on expected resource use and costs of delivery to the target population(s) Overall costs of interventions reported as well as costs of resource inputs Categorization of costs: capital vs. recurrent, fixed vs. variable Annualization of capital and fixed costs over the period of implementation [31] Adjusting estimated unit costs to the year of reported costs Converting costs into USD			Use of direct costs (exclude overheads) [50] Categorization of costs: Capital vs. recurrent, fixed vs. variable Annualization of capital and fixed costs over the period of implementation [31] (if applicable) No discounting [50] Converting costs into USD [31]	Use of financial costs/actual acquisition cost of intervention Data reported on expected resource use and costs of delivery to the target population(s) Overall costs of interventions reported as well as costs of resource inputs Categorization of costs: capital vs. recurrent, fixed vs. variable Annualization of capital and fixed costs over the period of implementation [31] Adjusting estimated unit costs to the year of reported costs Converting costs into USD
Modeling and analysis	*Use of decision*-*analytic model* Decision tree Markov model (individual-level/microsimulation or cohort), Discrete-event simulation Net benefit regression Extended cost-effectiveness analyses Compartmental model	Estimation of costs for alternatives by year of implementation; Analysis of key drivers of costs	Estimation of costs for single program by year of implementation; Analysis of key drivers of costs	Estimation of costs for single program by year of implementation; Division of cost by key outcome measures; Analysis of key drivers of costs	One of 3 analytic frameworks: BIA cost calculator, Condition-specific cohort, or Individual simulation model [50] Validation of computing framework and input data Estimation of costs for alternatives by year of implementation; Analysis of key drivers of costs	Application of TCO model or other simple excel based spreadsheet
Account for uncertainty	Recommended assessment of 3 types of uncertainty: *Structural uncertainty* introduced by the assumptions made *Source of values for parameter estimates* *Parameter precision*—uncertainty around the mean health and cost inputs in the model Gold standard is to conduct *Probabilistic sensitivity analysis*. This enables the uncertainty associated with parameters to be assessed simultaneously and incorporated in the results of the model	Threshold, univariate and multi-variate sensitivity analyses are most commonly used			*Parameter uncertainty* in the input values used *Structural uncertainty* introduced by the assumptions made in framing the BIA	Threshold, univariate and multi-variate sensitivity analyses are most commonly used Adjustment for optimization bias and risk exposure
Interpret and present results	Study parameters Summary of incremental costs and consequences Cost effectiveness plane Tornado diagram/threshold analyses Cost effectiveness acceptability curve Characterizing heterogeneity Discuss generalizability Describe underlying assumptions	Summary of unit costs Summary of aggregate and sub-category costs by activity and level of the health system for each year of implementation for each alternative	Summary of unit costs Summary of aggregate and sub-category costs by activity and level of the health system for each year of implementation		Design of the BIA reported Disaggregated and annualized BIA which demonstrates implications on Government and social insurance budgets; Households and out of pocket expenses; Third-party payers; and External donors [50] Both budget period resource use and costs should be presented	Summary of unit costs Summary of aggregate and sub-category costs by activity and level of the health system for each year of implementation
Ensure quality in reporting your evidence	Drummond Checklist [25] and/or CHEERS checklist screening [58]; MEEP principals for Economic Evaluation [31]	Reporting standards not available; select components of the CHEERS checklist can be applied			ISPOR principles of good practice [50]	Donor specific reporting standards

Synonymous with budget holder perspective

### Which type of analyses are right for me?

Stages 1–6 outline an optimal stage-based process for integrating economic and financial evaluations into mHealth programs. Figure 1 demonstrates the iterative nature of how these steps are intended to repeat at multiple points to generate evidence necessary to inform and catalyze the progression of an mHealth solution across stages of maturity from pre-prototype, prototype, pilot, to demonstration and ultimately scaling up, and similarly across stages of evaluation from efficacy, effectiveness to implementation science. Table 2 illustrates the relationship between many stage-based activities by highlighting differences and similarities in the content of each step required to execute them. Changes in the program design and underlying components inherent with implementation in different contexts and at increasing scale are likely to correspond to changes in both the costs and consequences, requiring repeated measures of effectiveness and value for money to ensure appropriate returns as maturity increases. Optimally, application of these stages would occur in a sequential linear process at each stage of maturity, drawing from data obtained in prior stages. In practice, finite resources, including technical expertise, coupled with limitations in timing of initiation may render completion of all stages infeasible. Accordingly, a five-step process helps prioritize to match stakeholders’ needs.

### Step 1. Define where the technology is in the stage of maturity and in the stage of evaluation

**Fig. 3:**
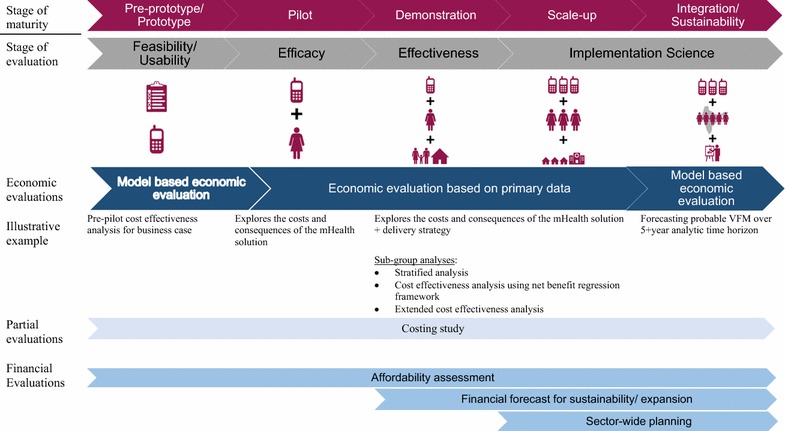
Linking the stages of maturity and evaluation with economic and financial evaluations (modified from [17])

A critical starting point in defining which economic and financial evaluation activities are right for you, lies in first defining where an mHealth solutions lies within its stages of maturity [17]. The stage of maturity is the continuum from pre-prototype and prototype, where mHealth solutions are in their earliest stages of evolution and testing, up to fully fledged integration into the health system at scale (Fig. 3). Closely aligned with the stage of development, is to identify the corresponding stage of evaluation. The stage of evaluation ranges from efficacy (implementation under controlled circumstances), effectiveness (implementation under real-world conditions), to implementation science (Assesses the uptake, integration and sustainability of evidence-based mHealth interventions) [17]. Figure 3 outlines the economic and financial evaluation activities indicated for each stage of maturity and stage of evaluation. While it is envisioned that Stages 1–6 be repeated at each stage of maturity as the mHealth solution moves along the continuum, the types of economic evaluations (model vs. trial based; CBA vs. CEA vs. CUA) undertaken will also be driven by larger study design considerations, available data, policy timelines, and technical expertise.

At all stages of maturity, a business case will help estimate value for money, refine planning for program design, implementation, management, and M&E. For mHealth solutions in the early stages of maturity, evaluation activities are likely to focus on the functionality, stability and usability of the technology and refining the implementation strategy. A full economic evaluation based on primary data is unlikely to be useful at this stage given the likely modifications which may occur in the technology and implementation strategies. However, a model-based economic evaluation that focuses on the value for money of expansion might (1) catalyse efforts to secure funding; (2) facilitate planning for piloting; (3) or allow decision-takers to rule out further action. If the stage of maturity increases and an mHealth solution progresses beyond a pre-pilot/pilot phase to a demonstration level of maturity, a full economic evaluation which focuses on primary data obtained through trial/program implementation is recommended. The feasibility of this depends on the study design and more specifically, availability of a comparator (whether modelled or based on primary data). However, if an economic evaluation is conducted and demonstrates value for money, efforts should then be undertaken to assess (1) the heterogeneity of program uptake and in turn costs and consequences across sub-groups (Stage 4); (2) financial costs for expansion/sustainability (Stage 5); (3) affordability (Stage 6). For mHealth programs at a demonstration level that do not have a comparator, a partial evaluation or costing study may be all that is feasible. While costing studies will not tell you the value of your mHealth program as compared to alternative resource uses, they will allow you to identify key drivers of costs and potential areas for cost savings, while setting the stage for affordability analyses. For programs within a scale-up or integration/sustainability stage of maturity, identifying a comparator may be challenging unless the nature of the rollout of the mHealth solution can be influenced to accommodate a quasi-experimental or stepped wedge study design. Where this is possible, the value for money of the scaled mHealth solution can be assessed drawing from primary data. Where a comparator is not feasible, a model-based economic evaluation or single-arm costing study may be executed and the primary emphasis shifted towards assessing the affordability to different budget holders in different contexts and/or creating a sustainable business model. The latter may be accompanied by broader system-wide assessments through application of modelling tools such as OneHealth.

### Step 2. What M&E activities are planned as part of the overarching mHealth program?

Most economic and financial evaluations are nested within larger program M&E activities. They overlap in data requirements, with economic analyses depending on effectiveness data obtained through overarching M&E, and require extensive support to execute, both from program managers and financial staff overseeing the program. The overarching study design for the evaluation, coupled with scope and content of M&E activities, helps to define the scope of economic analyses and identify opportunities for integrating costing analyses into the on-going M&E. Beyond drawing effectiveness data from the larger study, opportunities to incorporate questions on out of pocket payments to users, socioeconomic status and financial protection into planned or existing surveys may allow for more comprehensive perspectives to be considered and alternative sub-analyses to be undertaken. Further opportunities to ensure that program staff appropriately document key activities associated with program development, start-up and implementation will ensure that all components and resources used to execute the program are identified, measured and valued. The extent to which synergies can be identified between M&E and costing activities will help to minimize demands on finite project resources and optimize the scope and use of economic analyses to inform program implementation, expansion and sustainability.

### Step 3. Which evidence needs are appropriate for your mHealth solution?

A critical consideration in defining the scope of economic analyses lies in first considering how the data are intended to be used, by whom, and when. Full economic evaluations answer vital questions on the costs, benefits and comparative use of resources, while financial evaluations can estimate resource and financing requirements for expansion, and provide foundations for assessing affordability. If planned appropriately from the outset, adopting the full range of analyses highlighted in Stages 1–6 should be considered. In practice, this may be infeasible or cost-prohibitive, in which case, for mHealth solutions *with a comparator*, answering basic questions on value for money, heterogeneity in costs and consequences, and affordability should be prioritized. For mHealth solutions *without a comparator*, a model-based full economic evaluation may be possible, drawing on primary data on the program’s implementation costs. If this is not feasible, a partial evaluation or costing study may be undertaken. This can be followed by a basic estimate of expansion costs and affordability. However, in the absence of data on value for money that compares the mHealth solution to an alternative, it remains difficult to justify expansion. Given the likely changes to the program components associated with an mHealth solution’s maturation and/or its introduction to new contexts, one could argue that analyses from an early timepoint in an mHealth program’s implementation may not be generalizable to a more mature program. It is therefore important to appreciate the caveats underpinning economic evidence and where possible, advocate that analyses be repeated as maturity increases.

### Step 4. What is the time point of initiation for economic analyses?

The time point for starting economic analyses within a larger program funding and implementation cycle is a critical consideration in refining study objectives and activities. What is the timeline under which analyses must be completed? Has program implementation already begun? Many economic analyses are initiated after implementation, and many of M&E activities have been defined. These analyses may require the retrospective measurement of costs and effects and may be limited by the study design inherited and the availability of data and associated opportunities for primary data collection. While modelling is inherent in most economic analyses, primary collection of reliable and appropriate data, while challenging, is essential for capturing the full spectrum of costs and consequences. Economic analyses planned from a project’s inception allow for the prospective tracking of costs and consequences as implementation unfolds. Economic analyses that aim to estimate events into the future can occur either at a project’s end to catalyse expansion or at its inception, requiring analysts to hypothesize and model changes in how the program might look under a range of options in varied contexts.

### Step 5. Available resources

It is critical to assess the technical and financial resources available to conduct economic analyses. While most financial evaluations can be conducted by program staff with an accounting background, economic evaluations will require technical support from a health economist or someone with prior expertise conducting and publishing value for money analyses. The challenge with the latter lies not only in collecting, assembling, and analysing data on costs and consequences but in executing comprehensive uncertainty analyses which have become the gold standard for economic evaluations. Beyond requirements for technical support, it is important to ensure that sufficient resources have been set aside to support economic analyses and in particular primary data collection requirements. The latter may entail face to face structured interviews with respondents to capture data on costs and consequences not obtained through overarching monitoring and evaluation activities. In the absence of appropriate technical and financial planning, resource limitations can heavily impede the quality and rigor of economic analyses.

## Conclusions

This paper describes a stage-based process for integrating economic and financial evaluations into business case and M&E activities of mHealth solutions in LMICs. Where economic evaluations generate evidence on which programs represent the best value for money, financial evaluations can provide evidence on the financing required to initiate, sustain and/or expand programs as well as assess their affordability [52]. By highlighting synergies in the contents of economic and financials evaluation activities, we demonstrate how they can be implemented concurrently at multiple time points within the lifecycle of an mHealth solution to catalyze progression across stages of maturity. With proper planning and adequate resources, economic and financial evaluations can generate evidence essential to improve the allocation of finite resources, program planning, implementation, efficiency, effectiveness and sustainability.

## Abbreviations

BIA: budget impact analysis
CBA: cost benefit analysis
CEA: cost effectiveness analysis
CMA: cost minimization analysis
CUA: cost utility analysis
DALY: disability adjusted life years
ECEA: extended cost effectiveness analysis
ICER: incremental cost effectiveness ratios
LiST: Lives Saved Tool
LMICs: low and middle income countries
mHealth: mobile health
M&E: monitoring and evaluation
NBRF: net benefit regression framework
QALY: Quality Adjusted Life Years
RMNCH: reproductive, maternal, newborn, and child health
TCO: total cost of ownership

## Footnotes

1 Conceptual models help to identify and illustrate the relationships among factors (systemic, organizational, individual or other) that may influence the operation of an intervention and the successful achievement of the intervention’s goals [23].

2 Financial risk protection aims to ensure access to quality healthcare as needed without incurring financial hardship [36].
